# Cost comparison of phosphodiesterase type 5 inhibitors: rural vs urban New York State counties and online pharmacies

**DOI:** 10.1093/sexmed/qfaf031

**Published:** 2025-06-10

**Authors:** Sofia Maurina Di Scipio, Aaron Katz

**Affiliations:** Department of Urology, NYU Grossman Long Island School of Medicine, Mineola, NY 11501, United States; Department of Urology, NYU Grossman Long Island School of Medicine, Mineola, NY 11501, United States

**Keywords:** phosphodiesterase 5 inhibitors, erectile dysfunction, drug costs, rural population

## Abstract

**Background:**

Phosphodiesterase type 5 (PDE5) inhibitors are used to treat erectile dysfunction, but their cost can limit access.

**Aim:**

This study examines PDE5 inhibitors pricing and demographic data across rural and urban New York State (NYS) counties, as well as small, large, and online pharmacies.

**Methods:**

Prices from 133 chain pharmacies were collected from 15 randomly selected urban and 15 rural NYS counties. Counties without at least two large chain pharmacies were excluded. Data included small chains (*n* = 49), large chains (*n* = 84), rural (*n* = 57), urban (*n* = 76), and online pharmacies (*n* = 12). Prices for 20 tablets of sildenafil (100 mg) and tadalafil (20 mg) were gathered using GoodRx standard coupons. Price per unit for online pharmacies was calculated for quantities closest to 20 pills. Demographic data was sourced from the U.S. Census Bureau. This study was considered exempt from IRB review. Statistical analyses including *t*-tests, Wilcoxon rank-sum, and Kruskal–Wallis tests were performed using R Version 4.4.1 (2024-06-14).

**Outcomes:**

The cash price of the PDE5 inhibitors across various pharmacy chain types and county types.

**Results:**

Prices were lower in small chain pharmacies compared to larger chains (*P* < .001), but did not significantly differ between rural and urban counties (*P* > .6). Small chain rural pharmacies were cheaper than urban counterparts for sildenafil (*P* = .032). Online pharmacies offered the lowest and highest prices, with significant differences by chain type (*P* < .001) but not by county type (*P* > .100) for both drugs. Rural counties had a smaller Native Hawaiian (*P* = .001), Asian, Black/African American population and a larger White (*P* < .001) and American Indian population (*P* = .031). Median income was higher in urban counties (*P* = .010), but the percentage of the population without health insurance coverage did not differ (*P* = .177).

**Clinical Translation:**

This study aims to highlight the pricing variability of PDE5 inhibitors to help patients identify cost-effective options to circumvent potential financial barriers.

**Strengths and Limitations:**

This study was the first to examine PDE5 inhibitors pricing specifically within rural populations while also providing a comparative analysis of pricing differences between small and large pharmacy chains serving these communities. The study’s limitations include a relatively small sample size of rural and small chain pharmacies resulting in power levels of 75% and 69%, respectively, which may impact the generalizability of the findings.

**Conclusion:**

Enhancing drug price transparency for PDE5 inhibitors is vital for increasing access and pricing flexibility.

## Introduction

Phosphodiesterase type 5 (PDE5) inhibitors are competitive inhibitors of phosphodiesterase which were initially developed to treat pulmonary hypertension. However, their ability to enhance blood flow has led to their common use in managing erectile dysfunction (ED). In the United States, approximately 30 million men are negatively affected by ED.^1^ Phosphodiesterase type 5 inhibitors function by blocking phosphodiesterase from hydrolyzing cGMP to GMP, a process which causes cessation of smooth muscle relaxation.^2-4^ Without this hydrolysis, PDE5 inhibitors prolong the effects of cGMP resulting in vasodilation of penile arteries and sustained erections.^2-4^

Pricing for first generation PDE5 inhibitors, such as sildenafil and tadalafil, varies based on pharmacy type, dosage, quantity, and geographic location. For instance, significant price variability has been observed between large metropolitan areas with no correlation found between pricing and socioeconomic factors.^5^ Large pharmacy chains also tend to provide more affordable options than small chains pharmacies for the cash price of generic medications.^6^ In addition to traditional brick-and-mortar pharmacies, direct-to-consumer (DTC) platforms and online pharmacies have emerged as an alternative for patients seeking convenience and potential cost savings. While some online pharmacies advertise competitive pricing, prior research has shown that these platforms are often more expensive than chain pharmacies.^7-10^ However, the introduction of newer online platforms may offer affordable prices that challenge traditional pharmacies.

This study aimed to explore the following questions: How does PDE5 inhibitor pricing vary between rural and urban counties in New York State (NYS)? Do large chain pharmacies offer more affordable prices compared to small chain pharmacies and online pharmacies? We hypothesize that rural counties will have higher PDE5 inhibitor prices than urban areas. In addition, we expect large chain pharmacies to offer more affordable options compared to small chains and online pharmacies.

## Methods

This was a quasi-experimental observational study that randomly selected 15 urban and 15 rural NYS counties from a total of 62 NYS counties. This study was deemed exempt from IRB review since data was publicly available and de-identified. Counties were classified as rural or urban based on the definitions provided in the *State of Rural New York Report* by the Rural Housing Coalition of New York (2023).^11^ According to this report, rural counties were described as areas where 91%-100% of the population reside in communities with 25 000 residents or fewer. Urban counties were considered as areas where 0%-50% of the population reside in communities with 25 000 residents or fewer. Using these definitions, 35 counties were considered rural and 16 counties were considered to be urban. Chain pharmacies within the selected counties were identified by conducting a Google search for “pharmacies in [County Name], NY” and confirming their presence on the GoodRx website.

### Pharmacy categorization

Pharmacies were categorized as large chains if they were among the top 15 U.S. pharmacies ranked by total prescription revenues in 2023 based on the 2024 Economic Report on U.S. Pharmacies and Pharmacy Benefit Managers.^12^ Examples of large chain pharmacies include CVS, Walgreens, Walmart, and Rite Aid, which were prioritized when available. Small chain pharmacies were defined as those not appearing in the top 15 by total prescription revenue. Counties that did not have at least two large chain pharmacies were excluded from the study to ensure price comparisons included both large and small pharmacy chains in rural and urban areas. Since large chain pharmacies were more prevalent than small chains, this criterion allowed for a more balanced comparison without excluding too many counties that had fewer small chain pharmacies. A total of two rural counties (Hamilton and Allegany) were excluded in this study.

Online pharmacies and DTC platforms offering sildenafil and tadalafil were initially identified from a list of pharmacies derived from Forbes Health’s compilation of the Best Online Pharmacies of November 2024, which includes reputable and widely recognized platforms.^13^ Additional platforms were identified through targeted Google searches using terms such as “buy 100 mg sildenafil” and “buy 20 mg tadalafil” to ensure comprehensive coverage. Online pharmacies and DTC platforms were grouped together as both provide prescription medications directly to patients through online services. Online pharmacies were not assessed by county type since they are accessible nationwide, independent of geographic location. These pharmacies were included as an overall comparison to rural and urban pharmacies to evaluate their potential for providing the least expensive options. Pharmacies and platforms were included if they offered clear and accessible pricing information available on their website. For certain platforms, accounts were created to obtain exact pricing; however, websites requiring payment information or consultations to access pricing details were excluded from the analysis.

Prices for 20 tablets of sildenafil (100 mg) and tadalafil (20 mg) were collected for small and large chain pharmacies using GoodRx (https://www.goodrx.com) with standard coupons included. A quantity of 20 pills was selected rather than a monthly prescription to better reflect typical usage since PDE5 inhibitors for ED are usually taken on an as-needed basis. The price per unit (PPU) for the PDE5 inhibitors was calculated for quantities closest to 20 pills for online pharmacies. The data was collected in September 2024 and included 133 pharmacies with small chains (*n* = 49), large chains (*n* = 84), rural (*n* = 57), urban (*n* = 76), and online pharmacies (*n* = 12).

### Demographic data collection

Demographic data including median household income, the percentage of the population without health insurance coverage, and racial composition were obtained for each county from the U.S. Census Bureau.^14-16^ The data was collected in September 2024. Demographics were assessed to identify potential socioeconomic influences on drug pricing across county types.

### Statistical analysis

Median and average drug costs were calculated for each drug by county type (rural and urban) and for pharmacy type (large chain, small chain, and online). Interquartile ranges (IQRs) were additionally calculated for each drug. The normality of the data was tested with Shapiro–Wilk tests and non-parametric tests (Wilcoxon rank-sum tests for two groups and Kruskal–Wallis tests for three groups) were performed due to non-normal distributions. All tests were performed using a significance level of 0.05 and were two-sided. All statistical analyses and visualizations were generated using R Version 4.4.1 (2024-06-14) (R Core Team) with RStudio Version 2024.12.0.467 (Posit PBC) packages.^17-22^

## Results

**Figure 1:**
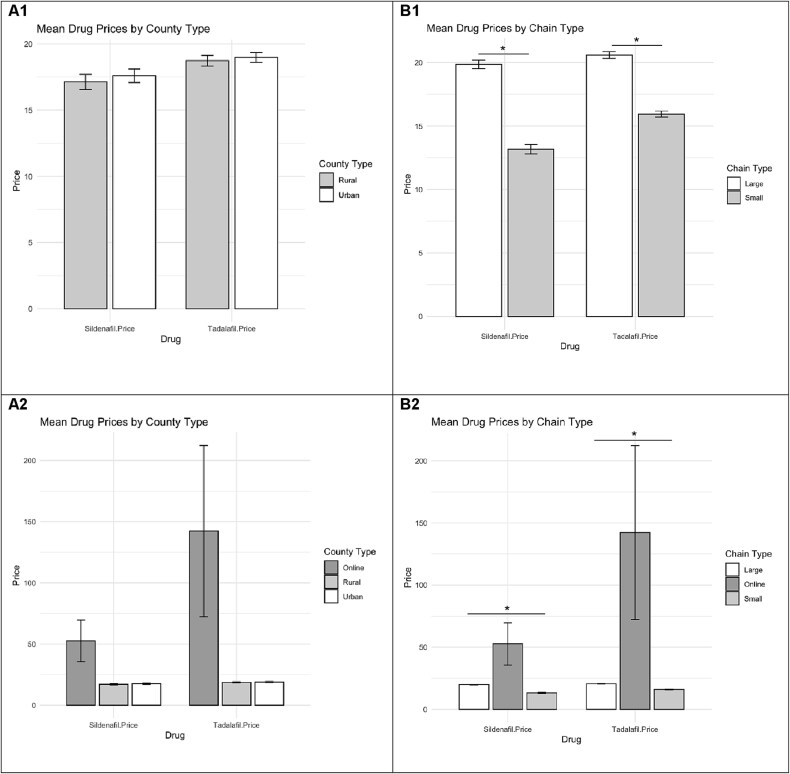
Mean costs for a 20 pill supply of phosphodiesterase type 5 (PDE5) inhibitors by county type (A1, A2), chain type (B1, B2), and with online pharmacies (A2, B2).

This study included 133 NYS pharmacies with 57 located in rural counties and 76 in urban counties (Table SS1). Fifteen rural and fifteen urban counties were included with a total of 2 rural counties excluded. Of these, 49 were small chain pharmacies and 84 were large chain pharmacies (Table 1). Additionally, 12 online pharmacies were reviewed. Prices of PDE5 inhibitors did not significantly differ between rural and urban pharmacies for sildenafil (*P* = .613) or tadalafil (*P* = .677) (Figure 1A1). The largest variation in prices was observed among online pharmacies which offered both the lowest and highest prices for sildenafil and tadalafil. For instance, the prices for 20 pills of sildenafil and tadalafil were $5.13 and $5.73 for Cost Plus Drugs and $200 and $880 for Roman. However, prices from online pharmacies were not significantly different by county type for sildenafil (*P* = .211) or tadalafil (*P* = .173) (Figure 1A2).

When comparing the price of PDE5 inhibitors by chain type, small chain pharmacies offered less expensive options for sildenafil (*P* < .001) and tadalafil (*P* < .001) compared to large chain pharmacies (Figure 1B1). Online pharmacies reported significantly higher mean and median prices than small and large chain pharmacies for sildenafil (*P* < .001) and tadalafil (*P* < .001) (Figure 1B2). Small chain rural pharmacies were also found to exhibit cheaper options than small chain urban pharmacies for sildenafil (*P* = .032), but not tadalafil (*P* = .636) (Table 2). However, no differences were found between large chain rural and large chain urban pharmacies for sildenafil (*P* = .500) or tadalafil (*P* = .224) (Table 2).

Demographic variables were additionally included for each NYS county in this study (Table 1). Rural counties had a smaller Native Hawaiian (*P* = .001), Asian, Black/African American population and a larger White (*P* < .001) and American Indian population (*P* = .031). Median income was higher in urban counties (*P* = .01) with a larger IQR (IQR = 24 009) compared to rural counties (IQR = 8786). The percentage of the population without health insurance coverage did not differ significantly by county type (*P* = .177).

## Discussion

Erectile dysfunction has been demonstrated to have negative biological and psychosocial impacts on the lives of 30 million American men.^1^ Although sildenafil and tadalafil are safe and effective treatments for ED, barriers to accessing these medications may impact compliance. In this study, 133 pharmacies and 12 online pharmacies were categorized by pharmacy type, small and large chain, and county type, rural and urban. Although some online pharmacies offered the most affordable prices, on average online pharmacies were significantly more expensive when compared by county and chain type. These findings were consistent with previous literature.^7-10^ Patients seeking these online alternatives are often motivated for a more cost-effective and convenient way to receive necessary medications.^7^^,^^23^ The increasing prevalence and favorable media coverage of these platforms have increased the appeal of DTCs.^10^ Even though DTC platforms are marketed as cost-effective and accessible, the for-profit aspect of these companies translates to higher costs for patients.^7^^,^^9^

While this study did not identify differences between county type prices for sildenafil or tadalafil, prices between chain types varied significantly for both medications (Table 1). Among small chain pharmacies, those in rural counties offered lower cash prices for sildenafil while no differences were observed among large chain pharmacies by county type (Table 2). It is possible that the role of pharmacy benefit managers (PBMs) in drug pricing for large chain pharmacies may explain the significantly higher prices found in this study. Large chain pharmacies rely on PBM-negotiated rebates, which can inflate the cash prices of these uninsured medications.^24^ In addition, the inefficiency of antitrust laws grants PBMs and large chain pharmacies more leeway to manipulate and artificially increase drug costs.^24^ If small chain pharmacies are not as entrenched by this network, they may display more drug pricing flexibility. The consequences of expensive PDE5 inhibitors are associated with medical nonadherence to ED treatments among other variables such as poor drug efficacy and decreased spontaneity.^25-29^

**Table 1:** Mean, median, and IQR costs for a 20-pill supply of sildenafil and tadalafil by county type (rural, urban), chain type (small, large), and online pharmacies. Demographic variables including median income, healthcare access, and race are compared for rural and urban counties. Statistically significant differences are indicated with asterisks (*).

	**Rural counties**	**Urban counties**	***P* value**	**Small chain**	**Large chain**	***P* value**	**Online**	**Online vs county type *P* value**	**Online vs chain type *P* value**
Mean (median; IQR)									
Pharmacy	*n* = 57	*n* = 76		*n* = 49	*n* = 84		*n* = 12		
Sildenafil ($)	17.13 (17.21; 6.13)	17.59 (17.21; 3.89)	.613	13.17 (12.63; 3.88)	19.86 (18.91; 7.38)	2.5e-17*	52.67 (33.00; 43.52)	0.211	2.5e-15*
Tadalafil ($)	18.73 (18.01; 4.77)	18.98 (18.53; 4.73)	.677	15.93 (15.37; 0.15)	20.59 (20.24; 6.18)	2.3e-17*	142.29 (52.85; 147.51)	0.173	3.9e-15*
Demographic variables: mean (median)									
Median Income (K)	65.0 (IQR: 8786)	77.0 (IQR: 24 009)	.010*						
Without Healthcare (%)	5.76 (4.3)	4.40 (3.9)	.177						
White (%)	87.21 (88.93)	57.99 (63.52)	1.6e-07*						
Black/African American (%)	3.00 (2.00)	14.35 (11.95)	5.0e-06*						
Asian (%)	1.00 (0.74)	8.48 (5.29)	3.4e-06*						
American Indian/Alaska Native (%)	1.13 (0.28)	0.66 (0.60)	.031*						
Native Hawaiian/Pacific Islander (%)	0.02 (0.03)	0.05 (0.05)	.001*						

Although median income was significantly higher in urban counties, medication prices did not vary by county type (Table 1). The larger IQR in urban counties indicates a more diverse mix of income levels with a maximum of $136 984 highlighting more affluent counties, and a minimum of $45 517 exhibiting lower-income areas. On the other hand, the rural counties studied had a tighter income distribution, with a maximum income of $80 372 and a minimum of $50 508 suggesting more homogenous economic conditions. This difference in median income may influence local pricing strategies through the lower sildenafil cash prices offered by small chain pharmacies in rural counties compared to urban areas (Table 2). This variation suggests that small chain pharmacies may be pricing sildenafil in accordance with the median income levels of their communities. Such pricing strategies could indicate efforts to address the needs of rural populations in improving medication affordability by accounting for local economic conditions. Further investigation is warranted to confirm and better understand this association.

The percentage of the population without health insurance coverage did not vary by county type. Cash prices were analyzed in this study because PDE5 inhibitors are often not covered by insurance or have limited coverage due to their classification as lifestyle medications. However, factors such as insurance type and healthcare access programs for low-income patients may impact their overall accessibility. Differences in insurance coverage between rural and urban communities could further impact the pricing of PDE5 inhibitors. For instance, rural communities tend to have lower median incomes which may affect patient access to healthcare. Rural areas also rely more on government-supported programs such as the New York Essential Plan and 340b Drug Pricing Program which allows for low-income patients to obtain medications at a reduced cost.^30^ This can offer insight into the more affordable pricing for sildenafil, in rural small chain pharmacies as these pharmacies may benefit from these programs and offer lower prices for rural communities. Further research is needed to understand the complex interactions between insurance coverage, pharmacy pricing trends, and disparities in access to ED medications.

**Table 2:** Mean, median, and IQR costs for a 20-pill supply of phosphodiesterase type 5 inhibitors by county type (rural, urban) and pharmacy chain type (small, large). Statistically significant differences are indicated with asterisks (*).

	**Small chain (*n* = 49)**			**Large chain (*n* = 84)**		
**Mean (median; IQR)**	**Rural (*n* = 18)**	**Urban (*n* = 31)**	***P* value**	**Rural (*n* = 39)**	**Urban (*n* = 45)**	***P* value**
Sildenafil ($ per 20 pills)	12.00 (11.29; 1.64)	13.85 (12.63; 3.73)	.032*	19.49 (18.91; 1.71)	20.17 (18.91; 7.38)	.500
Tadalafil ($ per 20 pills)	15.42 (15.37; 0.10)	16.24 (15.37; 0.15)	.636	20.26 (20.24; 2.23)	20.87 (20.24; 5.66)	.224

It is also important to examine the demographics of each county type within the context of healthcare access and disparities. Rural counties, characterized by higher proportions of White and American Indian populations and lower representation of Native Hawaiian, Asian, and Black/African American populations, face distinct barriers to healthcare access. These demographic differences are significant because they can shape healthcare needs and access to resources, emphasizing the importance of considering both racial and geographic factors when addressing disparities.^31^ For instance, differences in healthcare access among minority racial groups may intersect with geographic inequities, creating unique challenges in rural areas, especially because rural communities rely more heavily on independent pharmacies.^32^

Increased supply chain costs and declining market supply can further strain limited resources in rural communities. Although rural counties are often associated with reduced access to healthcare and prescription drugs, fluctuations in medication prices place added responsibility on physicians to stay informed about out-of-pocket costs to minimize financial strain on their patients.^5^^,^^33-35^ The findings of this study regarding the association between socioeconomic factors and the price of PDE5 inhibitors are consistent with previous research conducted by Levy et al.,^33^ but not with the findings of Mishra et al.^34^ or Nabavizadeh et al.^5^ The lower prices at small-chain pharmacies in rural areas highlight a potential strategy to address these disparities, but further research is needed to explore the interplay between racial demographics, geographic location, and medication access to develop more equitable solutions.

One limitation of this study is the small sample size for small chain pharmacies (49 out of 133) especially those in rural counties (18 out of 133). This limited sample size reduces the generalizability of our findings but also magnifies the extent of resource constraints faced by rural communities. Another limitation was the selection of a 20-pill quantity since a monthly supply could reduce the PPU, particularly on some DTC platforms that promote monthly bundles and subscriptions. Additionally, our initial search aimed to include independent pharmacies since prior studies have reported conflicting findings regarding the affordability of PDE5 inhibitors at independent pharmacies compared to traditional chain pharmacies.^7^^,^^33^^,^^34^

For instance, when comparing brand and generic PDE5 inhibitors, chain pharmacies were demonstrated to offer less expensive prices for generic medications compared to independent pharmacies.^5^ However, independent pharmacies were found to have less expensive PDE5 inhibitors in lower income neighborhoods compared to those in wealthier areas.^33^ Although socioeconomic factors are demonstrated to influence PDE5 inhibitor drug pricing in a study conducted by Levy et al.,^33^ this correlation was not consistent across the literature.^5^^,^^34^ Because of this variability, independent pharmacies were initially included in the dataset, but were ultimately excluded due the limited presence of these pharmacies in rural counties. Their exclusion from our analysis may have influenced our findings on pricing differences between rural and urban areas. In addition, the inclusion of more online pharmacies also posed challenges as many required prescriptions, payment information, or consultations before providing payment information.

Although no differences in pricing were found between rural and urban counties, differences in small and large chain pharmacies highlight an avenue to investigate the association between geographic location and different pharmacy chain types for PDE5 inhibitor medications. The tangible variations in cash prices further places the duty on physicians to guide their patients towards the most affordable prices to attenuate financial barriers to treatment.

## Conclusion

Erectile dysfunction is a physiological condition with tangible mental health and social implications for many men. Variations in PDE5 inhibitor pricing can create cost barriers that limit patients’ access to medications. This study identified an association between small and large chain pharmacy prices for sildenafil and tadalafil. The pricing trends observed in New York may reflect similar issues faced by patients and physicians in other regions which may provide insight into the price variation of PDE5 inhibitors in other states nationwide. These findings may help inform patients about the most cost-effective options for medications that are not traditionally covered by insurance, ultimately improving accessibility and adherence.

## Supplementary Material

Supplementary_Table_1_qfaf031
