# Supplementary material for: Cost comparison of phosphodiesterase type 5 inhibitors: rural vs urban New York State counties and online pharmacies
Source: Sex Med. 2025 Jun 10;13(3):qfaf031. doi: 10.1093/sexmed/qfaf031 (PMC12151535; doi:10.1093/sexmed/qfaf031)
Supplement: Supplementary_Table_1_qfaf031 [file supplementary_table_1_qfaf031.docx]

**Supplementary Table 1.** Pharmacy name, pharmacy type (small, large, online), county type (rural, urban) with county name included if applicable.

**Alt Text:** Table including pharmacy names, pharmacy type (small, large, online), county type (rural, urban), with county names if applicable.

| **Pharmacy Name** | **Pharmacy Type** | **County Type** | **County Name** |
| --- | --- | --- | --- |
| Walgreens | Large | Rural | Franklin |
| Walmart | Large | Rural | Franklin |
| Walgreens | Large | Rural | Oswego |
| Walmart | Large | Rural | Oswego |
| CVS | Large | Rural | Yates |
| Walgreens | Large | Rural | Yates |
| Rite Aid | Large | Rural | Yates |
| CVS | Large | Rural | Ulster |
| Walgreens | Large | Rural | Ulster |
| Walmart | Large | Rural | Ulster |
| Walgreens | Large | Rural | Wyoming |
| Walmart | Large | Rural | Wyoming |
| Rite Aid | Large | Rural | Wyoming |
| CVS | Large | Rural | Herkimer |
| Walgreens | Large | Rural | Herkimer |
| Walmart | Large | Rural | Herkimer |
| Walgreens | Large | Rural | Essex |
| Walmart | Large | Rural | Essex |
| CVS | Large | Rural | Schoharie |
| Walmart | Large | Rural | Schoharie |
| CVS | Large | Rural | Cattaraugus |
| Walgreens | Large | Rural | Cattaraugus |
| Walmart | Large | Rural | Cattaraugus |
| CVS | Large | Rural | Fulton |
| Walgreens | Large | Rural | Fulton |
| Walmart | Large | Rural | Fulton |
| CVS | Large | Rural | Cortland |
| Walgreens | Large | Rural | Cortland |
| Walmart | Large | Rural | Cortland |
| Walgreens | Large | Rural | Sullivan |
| Walmart | Large | Rural | Sullivan |
| CVS | Large | Rural | Ontario |
| Walgreens | Large | Rural | Ontario |
| Walmart | Large | Rural | Ontario |
| Walgreens | Large | Rural | St. Lawrence |
| Walmart | Large | Rural | St. Lawrence |
| CVS | Large | Rural | Greene |
| Walgreens | Large | Rural | Greene |
| Walmart | Large | Rural | Greene |
| CVS | Large | Urban | Erie |
| Walgreens | Large | Urban | Erie |
| Walmart | Large | Urban | Erie |
| CVS | Large | Urban | Nassau |
| Walgreens | Large | Urban | Nassau |
| Walmart | Large | Urban | Nassau |
| CVS | Large | Urban | Rockland |
| Walgreens | Large | Urban | Rockland |
| Walmart | Large | Urban | Rockland |
| CVS | Large | Urban | Kings |
| Walgreens | Large | Urban | Kings |
| Rite Aid | Large | Urban | Kings |
| CVS | Large | Urban | Orange |
| Walgreens | Large | Urban | Orange |
| Walmart | Large | Urban | Orange |
| CVS | Large | Urban | Queens |
| Walgreens | Large | Urban | Queens |
| Rite Aid | Large | Urban | Queens |
| CVS | Large | Urban | Onondaga |
| Walgreens | Large | Urban | Onondaga |
| Walmart | Large | Urban | Onondaga |
| CVS | Large | Urban | Schenectady |
| Walgreens | Large | Urban | Schenectady |
| Walmart | Large | Urban | Schenectady |
| CVS | Large | Urban | Richmond |
| Walgreens | Large | Urban | Richmond |
| Rite Aid | Large | Urban | Richmond |
| CVS | Large | Urban | Albany |
| Walgreens | Large | Urban | Albany |
| Walmart | Large | Urban | Albany |
| CVS | Large | Urban | Broome |
| Walgreens | Large | Urban | Broome |
| Walmart | Large | Urban | Broome |
| CVS | Large | Urban | Monroe |
| Walgreens | Large | Urban | Monroe |
| Walmart | Large | Urban | Monroe |
| CVS | Large | Urban | Suffolk |
| Walgreens | Large | Urban | Suffolk |
| Walmart | Large | Urban | Suffolk |
| CVS | Large | Urban | Bronx |
| Walgreens | Large | Urban | Bronx |
| Rite Aid | Large | Urban | Bronx |
| CVS | Large | Urban | New York |
| Walgreens | Large | Urban | New York |
| Rite Aid | Large | Urban | New York |
| Kinney Drugs | Small | Rural | Franklin |
| Kinney Drugs | Small | Rural | Oswego |
| Wegmans | Small | Rural | Oswego |
| Price Chopper | Small | Rural | Ulster |
| Tops Pharmacy | Small | Rural | Ulster |
| Kinney Drugs | Small | Rural | Herkimer |
| Price Chopper | Small | Rural | Herkimer |
| Kinney Drugs | Small | Rural | Essex |
| Price Chopper | Small | Rural | Essex |
| Price Chopper | Small | Rural | Schoharie |
| Price Chopper | Small | Rural | Fulton |
| Kinney Drugs | Small | Rural | Cortland |
| Price Chopper | Small | Rural | Cortland |
| ShopRite | Small | Rural | Sullivan |
| Wegmans | Small | Rural | Ontario |
| Kinney Drugs | Small | Rural | St. Lawrence |
| Price Chopper | Small | Rural | St. Lawrence |
| Price Chopper | Small | Rural | Greene |
| Tops Pharmacy | Small | Urban | Erie |
| Wegmans | Small | Urban | Erie |
| King Kullen | Small | Urban | Nassau |
| ShopRite | Small | Urban | Nassau |
| Duane Reade | Small | Urban | Nassau |
| ShopRite | Small | Urban | Rockland |
| Wegmans | Small | Urban | Kings |
| ShopRite | Small | Urban | Kings |
| Duane Reade | Small | Urban | Kings |
| Price Chopper | Small | Urban | Orange |
| ShopRite | Small | Urban | Orange |
| ShopRite | Small | Urban | Queens |
| Duane Reade | Small | Urban | Queens |
| Kinney Drugs | Small | Urban | Onondaga |
| Price Chopper | Small | Urban | Onondaga |
| Wegmans | Small | Urban | Onondaga |
| Price Chopper | Small | Urban | Schenectady |
| ShopRite | Small | Urban | Richmond |
| Duane Reade | Small | Urban | Richmond |
| Price Chopper | Small | Urban | Albany |
| Price Chopper | Small | Urban | Broome |
| Weis Pharmacy | Small | Urban | Broome |
| Wegmans | Small | Urban | Broome |
| Tops Pharmacy | Small | Urban | Monroe |
| Wegmans | Small | Urban | Monroe |
| King Kullen | Small | Urban | Suffolk |
| ShopRite | Small | Urban | Suffolk |
| Duane Reade | Small | Urban | Suffolk |
| ShopRite | Small | Urban | Bronx |
| Wegmans | Small | Urban | New York |
| Duane Reade | Small | Urban | New York |
| Hims | online | N/A | N/A |
| Roman | online | N/A | N/A |
| Cost Plus Drug | online | N/A | N/A |
| Costco Home Delivery | online | N/A | N/A |
| Amazon Pharmacy | online | N/A | N/A |
| Health Warehouse | online | N/A | N/A |
| Honeybee Health | online | N/A | N/A |
| Blink Health | online | N/A | N/A |
| Rx Outreach | online | N/A | N/A |
| DiRx | online | N/A | N/A |
| Treated | online | N/A | N/A |
| Male MD | online | N/A | N/A |
